# An Incidental Finding of a Gastrointestinal Stromal Tumor in a 62-Year-Old Male: A Case Report

**DOI:** 10.7759/cureus.31097

**Published:** 2022-11-04

**Authors:** Balakrishnan Kamaraj, Ruthwik Duvuru, Mohammed Afsharhussain Hithayathulla, Kaargil Puliyadi Rishi, Gowthami Sai Kogilatota Jagirdhar

**Affiliations:** 1 Medicine, Madurai Medical College, Madurai, IND; 2 Medicine, Mohammed Bin Rashid University of Medicine and Health Sciences, Dubai, ARE; 3 Internal Medicine, Saint Michael's Medical Center, Newark, USA

**Keywords:** immunohistochemistry (ihc), kit, indeterminate abdominal mass, hernia, gastrointestinal stromal tumor (gist)

## Abstract

Gastrointestinal stromal tumors (GISTs) are uncommon GI tract cancers that develop from immature mesenchymal cells. It might be difficult to get an early diagnosis of people with small bowel GISTs, which can cause delays in therapy. We present here a case of a 62-year-old male with an incidental finding of a small intestine GIST during the workup for umbilical hernia. He presented with swelling above the umbilicus for the past six months that was progressive in nature and not associated with pain. Computer tomography (CT) of the abdomen with intravenous contrast revealed a heterogeneously enhancing mass lesion in the left paraumbilical intraperitoneal region, and immunohistochemistry results of the CT-guided biopsy showed a GIST. The patient underwent excision of the tumor with segmental resection and anastomosis, and supraumbilical hernia repair. Chemotherapy (imatinib for three years) after suture removal was planned for him.

## Introduction

Gastrointestinal stromal tumors (GISTs) are rare mesenchymal tumors with characteristics of spindle cells, epithelioid cells, or occasionally pleomorphic cells. They are believed to be arising from Cajal's interstitial cells. Around 80% of GISTs are due to a mutation in the KIT gene or platelet-derived growth factor receptor alpha (PDGFRA) polypeptide, leading to tumor growth [1]. GISTs occur mainly in the stomach (60% of the cases), followed by the small intestine (30%) and colon. These tumors are generally diagnosed from the age of 50 to 70 with no gender predisposition [1,2]. The annual incidence that has been documented varies by region: in the United States, there are 3.2-6.8 cases per million people; in Europe, there are 2.1-14.5 cases per million people; and in Asia, there are 11.3-19.7 cases per million people [3].

Among these limited cases, 70% of the patients exhibit signs and symptoms that are more closely tied to the tumor's location. The most frequent symptoms are generalized stomach discomfort, bleeding, nausea, vomiting, abdominal distension, early satiety, abdominal pain, and rarely, a palpable abdominal mass [4]. Imaging investigations such as computed tomography (CT) and endoscopic ultrasound are used in diagnosing the disease; however, a tumor biopsy before surgery is generally not advised due to the risk of rupture. Contrast-enhanced CT is the preferred investigation for patients with symptoms of abdominal mass, and endoscopic ultrasounds are used with patients having gastrointestinal bleeding [5]. Laparoscopic surgical resection is the gold standard for treating GISTs, but open laparotomies are preferred if the patient is in an unstable condition. Imatinib is a preferred drug for the adjuvant treatment of tumors [6]. Total resection is possible in about 85% of patients, and 50% experience recurrence after complete resection. The median time for recurrence following the resection of a primary high-risk GIST is two years, and the five-year survival rate is roughly 50% [7].

## Case presentation

**Figure 1:**
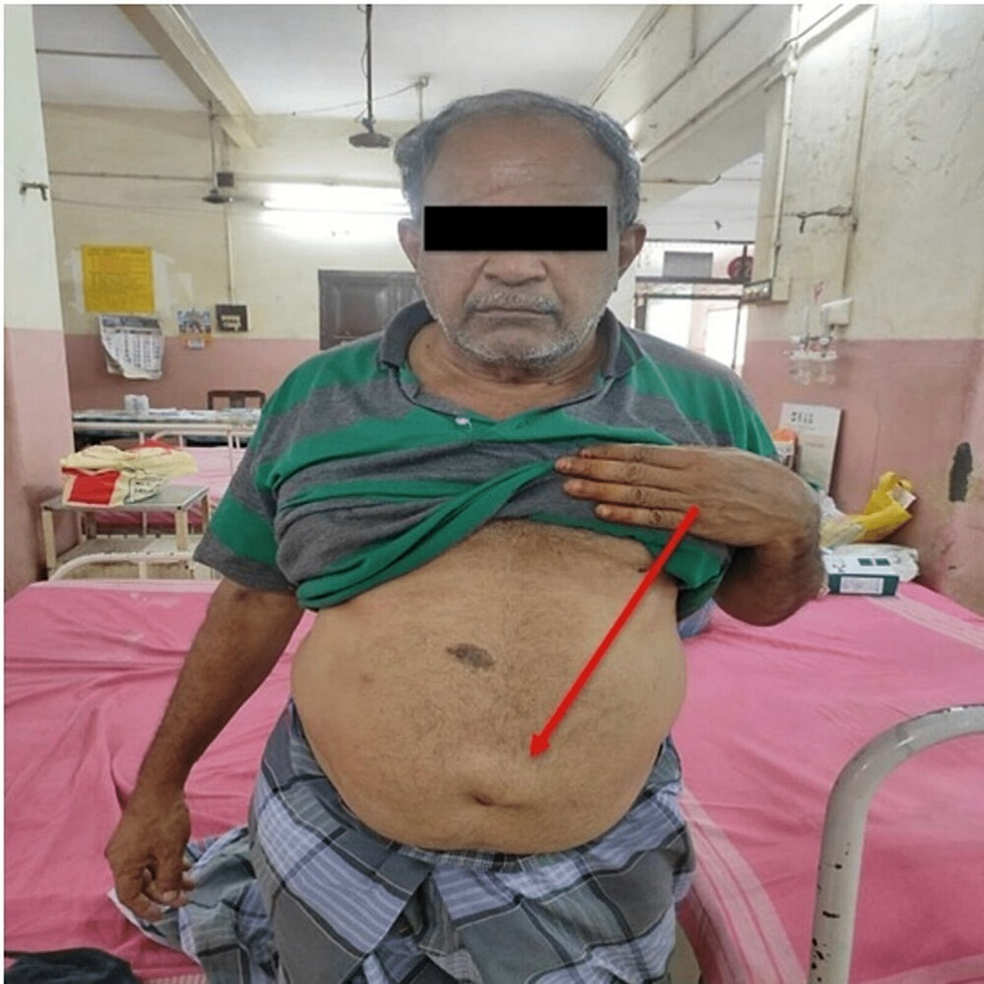
The supraumbilical swelling (arrow) that was later diagnosed to be an uncomplicated hernia

A 62-year-old male presented to the department of surgery with swelling above the umbilicus for the past six months, which was progressive in nature and not associated with pain (Figure 1). He denied any associated symptoms of nausea and vomiting. The patient denied any family history of GI disorders. The patient had a history of left-side open hernioplasty done 22 years ago at a private hospital and a laparoscopic omental patch repair done two years ago. The patient used to have a mixed diet, had regular bowel and bladder habits, did not drink alcohol and was a non-smoker. Excluding a palpable, non-tender supraumblical mass of 3 x 3 cm, the rest of the general and systematic examination was unremarkable. The palpable mass was soft, mobile, and reducible with a positive cough impulse. Laboratory findings were normal, including blood chemistries, routine blood tests, coagulation profile, tumor markers, ECG, and echocardiogram.

**Figure 2:**
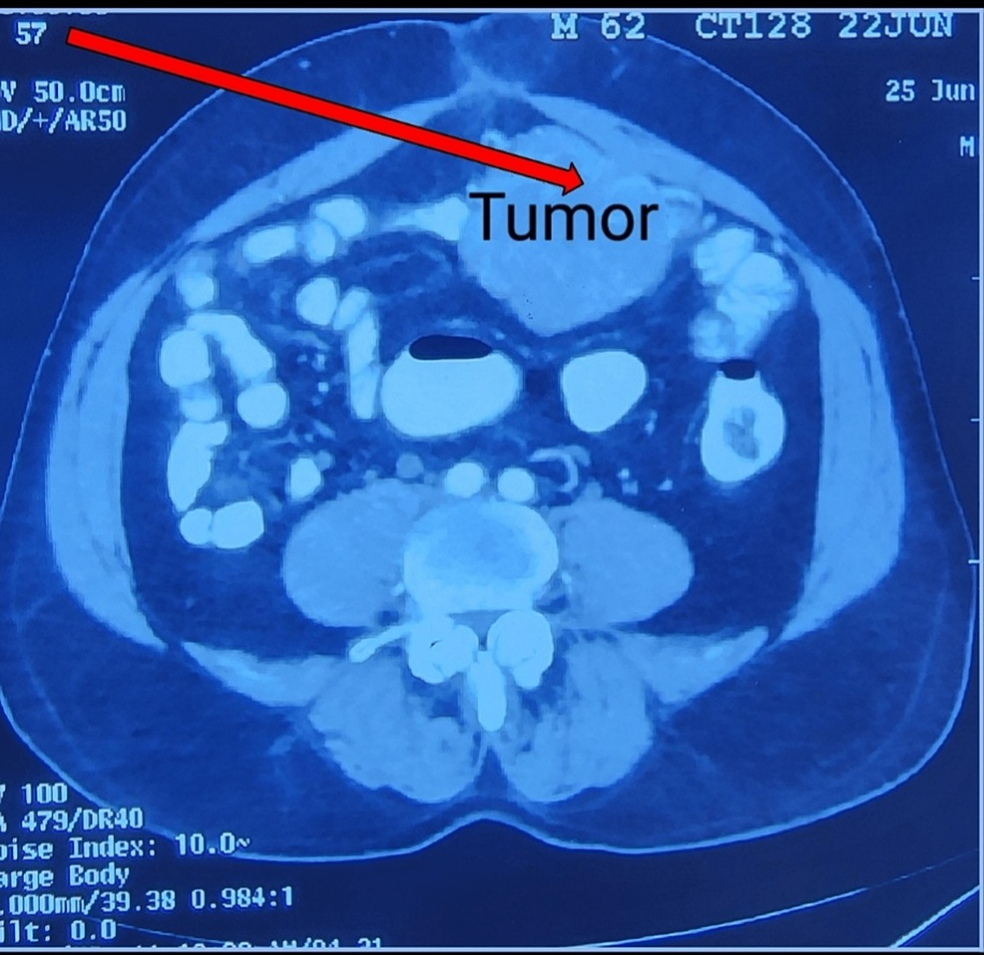
A dense soft tissue lesion (arrow) adherent to the terminal ileum loop in the midline

Ultrasonography of the abdomen and pelvis revealed a supraumbilical hernia of 1.4 cm in the longest axis with no concurrent obstruction containing adipose tissue. Abdominal CT without contrast revealed a supraumbilical hernia containing omentum and a dense, soft tissue lesion measuring 10.2 x 5.6 cm adherent to the terminal ileum loop in the midline. Due to the need for further evaluation and clinical correlation, abdominal CT with contrast was done that revealed an heterogeneously enhancing mass lesion approximately 8.2 x 7 x 9 cm in size in the left paraumbilical intraperitoneal region (Figure 2) with the loss of fat plane between the left anterior parietal wall and adherent to adjacent bowel loops, another 5 x 0.8 cm nodular lesion in left adrenal, suggestive of metastasis. Laboratory and radiological investigations gave us a differential diagnosis of the GIST and/or peritoneal mesothelioma.

**Figure 3:**
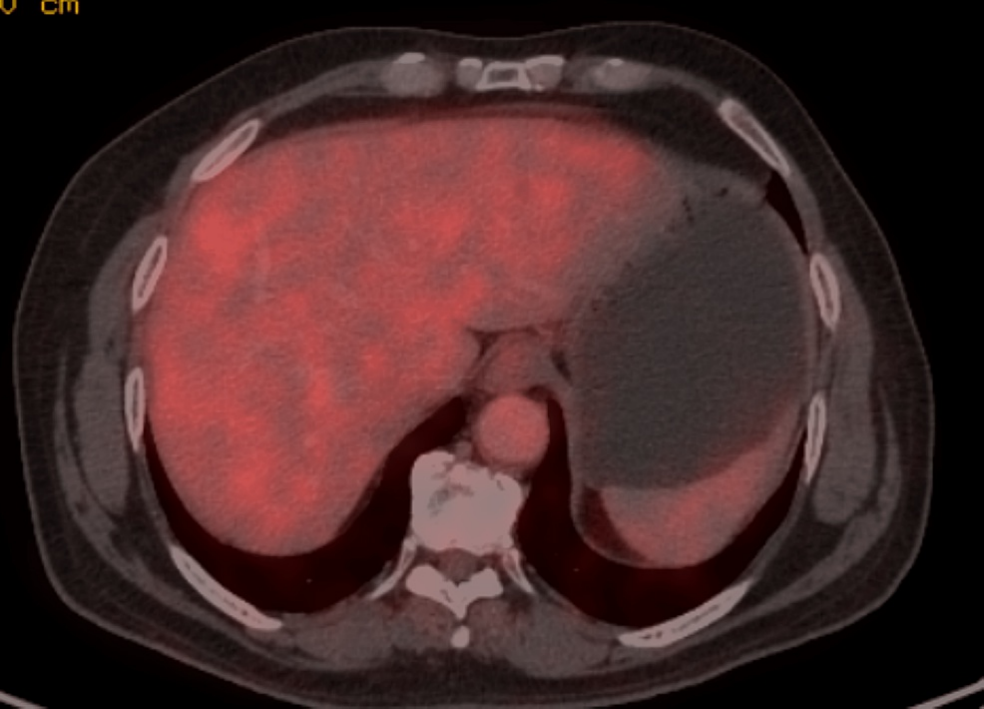
A normal positron emission tomography (PET) scan with no signs of liver metastasis

To confirm a diagnosis, the patient underwent a CT-guided biopsy, and microscopic findings revealed linear islands of sarcomatoid bundles of spindle-out cells with increased cellularity and occasional areas showing mitotic activity. Some areas showed attempted nuclear palisading. There were no areas of tumor necrosis in the sections studied. Following the CT-guided biopsy, our differential still remained to be GIST (neuronal type) or mesothelioma (fibrous type). The patient was later transferred to the surgical oncology unit for further investigation. A positron emission tomography (PET) scan revealed a metabolically active soft tissue mass noted in the midline of the lower abdomen closely abutting the jejunal and ileal loops (Figure 3). No evidence of metabolically active disease was noted elsewhere. The immunohistochemistry marker study revealed CD117 to be negative and Ki67 5%; this led us to the most likely diagnosis of small intestinal GIST, a spindle cell neoplasm.

**Figure 4:**
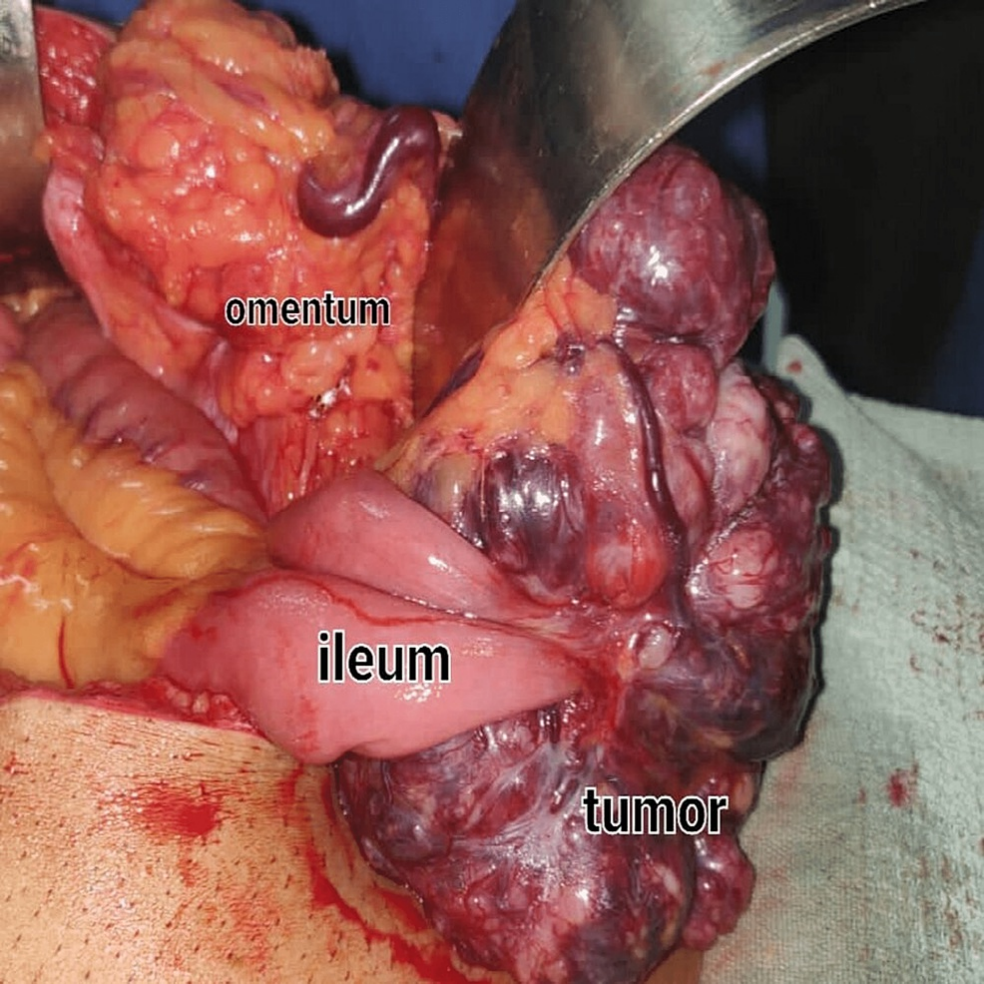
The exophytic GIST (10 x 8 cm) away from the antimesenteric border of the proximal ileum GIST, gastrointestinal stromal tumor

Upon receiving consent, the patient was prepared for the tumor excision with segmental resection, anastomosis, and supraumbilical hernia repair. Gross surgical findings included a 10 x 8 cm exophytic GIST away from the antimesenteric border of the proximal ileum and approximately 50 cm proximal to the ileocecal junction (Figure 4). The omentum burst through a 2 x 1 cm defect in the supraumbilical hernia. The immunohistochemical studies on the excised mass found DOG1 to be positive, calretinin to be negative, and S100 to be negative. The pathologist's impression was a gastrointestinal stromal tumor, which was a spindle cell type in the ileum part of the small intestine. The tumor was a high grade of stage pT3. Postoperatively the patient had normal vitals and was restricted to a high protein diet and given cephalexin 250 mg, paracetamol 500 mg, ranitidine 100 mg, metronidazole 200 mg, vitamin C, vitamin B complex, and nebulization. At the postoperative follow-up, the wound was well healed, sutures were removed and no high-risk factors were present.

## Discussion

Gastrointestinal stromal tumors are submucosal lesions that can occur anywhere in the gastrointestinal tract; however, nearly two-thirds of them occur in the stomach. Most frequently growing exophytically, these lesions can also occur endophytically with sizes ranging from 1 to 40 cm in diameter, with men slightly having a higher predisposition [8].

Although rare, GISTs have a gamut of clinical presentations, from abdominal pain and bloating to GI hemorrhage with necrosis and ulceration of the overlying mucosa. The go-to radiology scan is CT angiography for identifying the source of GI bleeding, thus only aiding in the diagnosis of GIST. Other tests, such as colonoscopy and gastroscope, can only identify bleeding but not the source. These tumors are managed through a microscopically margin negative resection, with no lymphadenopathy since these tumors do not tend to metastasize to lymph nodes [9]. GIST patients, with several risk stratification systems, including the National Institutes of Health (NIH)-Fletcher staging system, the NIH-Miettinen criteria, the Armed Forces Institute of Pathology (AFIP) risk criteria, and the Modified NIH criteria, are advised to assess the risk of recurrence, tumor size, mitosis count, and/or tumor site, as well as tumor rupture [10]. However, there are no generally acknowledged criteria of risk assessment for adjuvant therapy [10]. Several trials, such as the American College of Surgeons Oncology Group (ACOSOG) phase 2 Z9000, assessed the safety and efficacy of one year of adjuvant imatinib and reported one-year recurrence-free survival (RFS) at 96% and five-year overall survival rates at 83% [11]. Phase 3 of the ACOSOG trial reported one-year RFS at 98% in the imatinib arm compared to 83% in the placebo arm (95% confidence interval, 0.22 to 0.53; p<0.0001) [12].

Our patient presented with a supraumbilical hernia and no other symptoms associated with the GIST and therefore had to undergo extensive testing to determine the diagnosis and plan further management. Several papers have described incidental findings of large asymptomatic GISTs [13]. Our case highlights the importance of rigorous testing of the incidental lesions in the abdomen with radiological imaging as GISTS are one of the most common mesenchymal neoplasms of the gastrointestinal tract, and also to prevent a tumor that otherwise has a good prognosis when occurring in the stomach.

## Conclusions

We believe that our case report contributes to offering surgeons, gastroenterologists, and oncologists the prognostic data and outcomes in small intestinal GIST patients. Diagnosing a large asymptomatic GIST is possible, and given the malignant potential, early diagnosis and treatment are crucial. The histologic findings and genetic profile can potentially help in determining prognosis.
